# Decreased inpatient psychiatric admissions with telepsychiatry use during the COVID-19 pandemic

**DOI:** 10.3389/fpsyt.2023.1172019

**Published:** 2023-06-07

**Authors:** Brendon Lin, Anna Costakis, Majnu John, Howard Linder

**Affiliations:** ^1^Donald and Barbara Zucker School of Medicine at Hofstra/Northwell, Hempstead, NY, United States; ^2^Zucker Hillside Hospital, Northwell Health, Glen Oaks, NY, United States; ^3^Feinstein Institutes for Medical Research, Northwell Health, Manhasset, NY, United States

**Keywords:** telepsychiatry, psychiatric hospitalization, outpatient psychiatry, COVID-19 pandemic, hospitalization rate

## Abstract

**Objective:**

This study examines the quality of care provided through telepsychiatry by comparing psychiatric hospitalization rates among patients receiving in-person psychiatric care prior to the COVID-19 pandemic with rates among patients receiving virtual psychiatric care during the COVID-19 pandemic.

**Methods:**

Mental health-related hospitalization rates among patients enrolled in a large academic hospital’s outpatient psychiatry programs between March 1, 2018 and February 28, 2022 were retrospectively analyzed. Four time periods were created, spanning March 1 to February 28 of the following year. Demographic and clinical data were collected from the electronic health record, and descriptive statistics were calculated. Change in hospitalization rate between time periods was evaluated using McNemar’s test.

**Results:**

In the 2018 time period, 7.38% of all enrolled patients were hospitalized, compared to 7.70% hospitalized in the 2019 period, 5.74% in the 2020 period, and 5.38% in the 2021 period. Patients enrolled in both the 2018 and the 2019 periods saw no difference in hospitalization rate between the 2 years (2.93% in 2018, 2.83% in 2019; *p* = 0.830); patients enrolled in both 2019 and 2020 saw significantly lower hospitalization rates in 2020 (5.47% in 2019, 4.58% in 2020; *p* = 0.022); and patients enrolled in both 2020 and 2021 saw no difference (3.34% in 2020, 3.23% in 2021; *p* = 0.777).

**Conclusion:**

Psychiatric hospitalization rates significantly decreased between the 2019 and the 2020 periods, suggesting a decrease in admissions associated with adoption of telepsychiatry. Future research should differentiate the roles played by telepsychiatry and COVID-19-related factors in reducing hospitalization rates during the pandemic.

## Introduction

Over the past decade, telehealth has become an increasingly common modality for the delivery of healthcare (1, 2). This trend has been especially true in psychiatry, which has historically seen higher rates of telehealth usage compared to other healthcare specialties (2, 3). The high incidence of telehealth in psychiatry can in part be attributed to benefits such as increased attendance and patient convenience (4), as well as its cost-effectiveness (5). It has also been found to produce similar patient health outcomes as in-person psychiatric care (6–9), along with success in building rapport and establishing a therapeutic alliance (10–12). Telepsychiatry has been especially effective in the delivery of psychotherapy (5, 13), including for the treatment of depressive disorders (14) and eating disorders (10), and in providing group therapy (4, 15).

Prior to the COVID-19 pandemic, telepsychiatry was not widely used in the public health sector, in part due to regulatory restrictions. With the onset of the COVID-19 pandemic and public health-related restrictive measures, many regulatory restrictions were lifted, leading to telehealth becoming increasingly utilized across a wide range of healthcare specialties, including psychiatry (16–19). This sudden increase in telepsychiatry usage may have had significant consequences for patients’ mental health, due to potential disruptions to their access to and quality of care. Additionally, providers may struggle to adapt to this new modality. Understanding the impact of COVID-19 on the care delivered in psychiatry is of particular importance, since the COVID-19 pandemic and its associated restrictive measures have resulted in a greater national mental health burden (20–26).

Thus far, the use of telepsychiatry during the COVID-19 pandemic has been found to be largely satisfactory to providers (27–31), with providers noting its high acceptability, feasibility, and appropriateness (32). Noted benefits of telepsychiatry compared to in-person care include reduced viral transmission (33), increased patient attendance (33, 34), increased convenience for both providers and patients (27), and a reduction in logistical barriers (33, 35). Disadvantages include the risk of worsening preexisting disparities in healthcare access (33, 36–38), as well as increased challenges for older patients (27, 37, 38). Compared to in-person care delivered prior to the COVID-19 pandemic, care delivered virtually has yielded a similar reduction in patient symptomatology (34, 39). This finding was seen in telehealth delivered for both intensive outpatient treatment (39, 40) and for the partial hospital level of care (39–41), though the virtual partial hospital level of care was associated with a longer duration of treatment than its in-person counterpart (39, 41). Patient satisfaction and change in patient symptomatology were similar between virtual and in-person care for patients being treated for eating disorders (42–44) and for borderline personality disorder (45). Patients with obsessive-compulsive disorder also saw a similar change in their symptomatology (40), while patients with anxiety disorders saw a greater improvement in their symptoms than patients receiving in-person care (46).

One aspect of patient mental health that has received little attention is the rate of mental health-related hospitalizations during the COVID-19 pandemic, especially as compared to before the onset of the pandemic. The rate of mental health-related hospitalizations serves as a useful estimate both for changes in patient health outcomes following virtual psychiatric care, as well as for the severity of symptoms experienced by patients during the COVID-19 pandemic, as patients would only be hospitalized if their symptoms were sufficiently severe. In the initial months immediately following COVID-19-related restrictive measures, there was a reduction in total mental health-related emergency department visits and hospitalizations as compared to before the pandemic (47–50). There were fewer hospitalizations related to psychosis (51), schizophrenia (52, 53), and suicidality (54), but an increase in hospitalizations for anxiety and depressive disorders (47, 48) and substance use disorders (55). No differences were noted regarding the proportion of patients hospitalized following emergency department visit based on presenting condition (49, 56). By 6 months after the onset of COVID-19-related restrictive measures, emergency department visits, and hospitalizations related to psychosis (51), schizophrenia, and bipolar I disorder (52) returned closer to their pre-pandemic rates.

However, despite the evidence that has been presented thus far, it is difficult to ascertain the overall rates of mental health-related emergency department visits and hospitalizations during the COVID-19 pandemic. There have been few articles discussing this topic, with many of them presenting findings from only the early stages of the pandemic. It is challenging to extrapolate the quality of telepsychiatry care using these limited data, as, in the initial months following the imposition of restrictive measures, fear of contracting COVID-19 may have influenced the rate of emergency department visits as much as any change in the quality of psychiatric care being delivered.

In this retrospective study, we aim to address the gap in the literature by comparing mental health-related hospitalization rates before the COVID-19 pandemic with rates following the imposition of restrictive measures. We examine a population of longitudinal outpatients in a large academic hospital who first entered psychiatric treatment prior to the widespread adoption of telepsychiatry in March 2020, comparing their rates of hospitalization while receiving in-person care to their rates of hospitalization while receiving virtual care. We hypothesize that hospitalization rates during the in-person care period will not be significantly different from rates during the virtual care period, given the previously noted similarities between the two modalities in terms of change in patient symptomatology and patient satisfaction with care. This study will provide an important update to mental health-related hospitalization data during the COVID-19 pandemic, while also using hospitalization rate as a proxy to assess the quality of telepsychiatric care as compared to in-person psychiatric care. The findings from this study will be beneficial in evaluating the role of telepsychiatry in mental healthcare in the post-pandemic world.

## Materials and methods

We examine psychiatric hospitalization rates between March 1, 2018 and February 28, 2022. This 4-year period was divided into four individual time periods spanning March 1 of 1 year until the end of February in the following year (i.e., the 2018 time period encompasses March 1, 2018 through February 28, 2019). Patients enrolled in any of Zucker Hillside Hospital’s various outpatient psychiatry programs between March 1, 2018 and February 28, 2022 were eligible for consideration in this study, with the exception of patients enrolled in substance use disorder programs or partial hospitalization hospitals. We only considered patients who were enrolled in an outpatient program for the entirety of at least one time period, without interruption. Characteristics of patients in each of the four time periods, including gender, outpatient program type, diagnosis, and insurance coverage, are shown in Table 1.

**TABLE 1:** Characteristics of enrolled outpatients by time period.

Characteristic	2018		2019		2020		2021	
	*N*	%	*N*	%	*N*	%	*N*	%
**Gender**								
Male	4,265	42.7	4,396	43.1	4,361	41.3	4,515	39.8
Female	5,735	57.4	5,806	56.9	6,197	58.7	6,826	60.2
**Clinic type**								
Child clinics	2,056	20.6	2,172	21.3	2,314	21.9	2,477	21.9
Adult ambulatory clinics	5,755	57.6	5,923	58.1	6,119	58.0	6,535	57.7
Geriatric clinics	2,189	21.9	2,107	20.7	2,125	20.1	2,309	20.4
**Diagnosis**								
Anxiety disorders	995	10.0	1,050	10.3	1,177	11.1	1,348	11.9
Bipolar and related disorders	1,184	11.8	1,242	12.2	1,207	11.4	1,323	11.7
Depressive disorders	3,304	33.0	3,403	33.4	3,453	32.7	3,649	32.2
Disruptive, impulse control, and conduct disorders	29	0.3	34	0.3	38	0.4	47	0.4
Dissociative disorders	9	0.1	7	0.1	9	0.1	33	0.3
Feeding and eating disorders	27	0.3	25	0.2	26	0.2	39	0.3
Neurocognitive disorders	477	4.8	395	3.9	457	4.3	406	3.6
Neurodevelopmental disorders	822	8.2	886	8.7	913	8.6	933	8.2
Obsessive-compulsive and related disorders	419	4.2	472	4.6	533	5.0	652	5.8
Paraphilic disorders	10	0.1	12	0.1	11	0.1	9	0.1
Personality disorders	139	1.4	119	1.2	114	1.1	122	1.1
Schizophrenia spectrum and other psychotic disorders	2,197	22.0	2,155	21.1	2,185	20.7	2,269	20.0
Somatic symptom and related disorders	15	0.2	17	0.2	25	0.2	25	0.2
Trauma- and stressor-related disorders	371	3.7	382	3.7	410	3.9	466	4.1
**Insurance**								
Medicare	2,018	20.2	1,898	18.6	1,862	17.6	1,953	17.3
Managed medicare	1,047	10.5	1,065	10.4	1,059	10.0	1,153	10.2
Medicaid	206	2.1	174	1.7	156	1.5	154	1.4
Managed medicaid	2,283	22.8	2,352	23.1	2,526	23.9	2,819	24.9
Health maintenance organization (HMO)	3,913	39.1	4,192	41.1	4,435	42.0	4,720	41.7
Commercial	240	2.4	228	2.2	248	2.3	246	2.2
Self-pay	294	2.9	293	2.9	272	2.6	276	2.4

All patients considered for this study are included in individual year analysis. Here, we examine hospitalization rates during individual time periods among patients enrolled for the entirety of that time period. Hospitalization counts were determined through retrospectively querying the electronic medical record for patients enrolled in an outpatient psychiatry program who simultaneously were admitted into the Zucker Hillside Hospital inpatient service. Descriptive statistics were generated pertaining to hospitalization rate within individual time periods, including percentage of enrolled patients who were hospitalized and mean hospitalizations per enrolled patient.

Patients who were enrolled in care for any two consecutive time periods without interruption were additionally considered in paired analysis. Here, hospitalization counts and hospitalization rates are compared between the 2 years in the analysis. Descriptive statistics pertaining to hospitalization rates were generated for each of the 2 years. McNemar’s test was performed to determine significance of change in the proportion of patients who were hospitalized between the 2 years.

Institutional review board approval was waived.

## Results

### Individual year analysis

In the 2018 period, encompassing March 1, 2018 through February 28, 2019, there were a total of 10,000 patients enrolled. A total of 738 unique patients were hospitalized, accounting for 7.38% of the enrolled patient population. There were a total of 1,001 hospitalizations, representing a rate of 1.36 hospitalizations per hospitalized patient.

In the 2019 period, encompassing March 1, 2019 through February 29, 2020, there were a total of 10,202 patients enrolled. A total of 786 unique patients were hospitalized, accounting for 7.70% of the enrolled patient population. There were a total of 1,076 hospitalizations, representing a rate of 1.37 hospitalizations per hospitalized patient. Between the 2018 period and the 2019 period, the proportion of enrolled patients who were hospitalized increased (7.38% in 2018 to 7.70% in 2019), with a slight increase in the hospitalization rate (1.36 in 2018 to 1.37 in 2019).

In the 2020 period, encompassing March 1, 2020 through February 28, 2021, there were a total of 10,558 patients enrolled. A total of 606 unique patients were hospitalized, accounting for 5.74% of the enrolled patient population. There were a total of 811 hospitalizations, representing a rate of 1.33 hospitalizations per hospitalized patient. Between the 2019 period and the 2020 period, the proportion of enrolled patients who were hospitalized decreased (7.70% in 2019 to 5.74% in 2020), with a slight decrease in the hospitalization rate (1.37 in 2019 to 1.33 in 2020).

In the 2021 period, encompassing March 1, 2021 through February 28, 2022, there were a total of 11,341 patients enrolled. A total of 611 unique patients were hospitalized, accounting for 5.38% of the enrolled patient population. There were a total of 821 hospitalizations, representing a rate of 1.34 hospitalizations per hospitalized patient. Between the 2020 period and the 2021 period, the proportion of enrolled patients who were hospitalized decreased (5.74% in 2020 to 5.38% in 2021), with a slight increase in the hospitalization rate (1.33 in 2020 to 1.34 in 2021).

### Paired analysis

**FIGURE 1:**
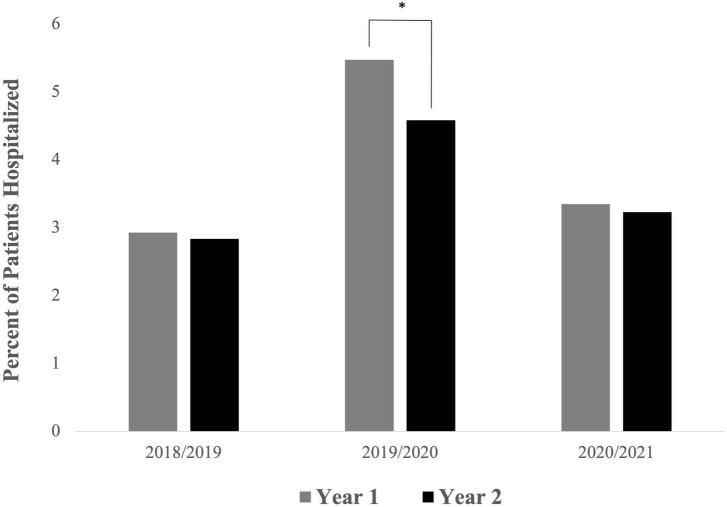
Percent of patients with mental health-related hospitalizations per year among patients enrolled in outpatient psychiatric care for two consecutive years. Patients enrolled in both the 2019 and 2020 time periods saw a significant decline in hospitalization rate between Year 1 and Year 2 [*p* = 0.022; OR = 1.213, 95% CI (1.009, 1.458)], whereas the other two paired time periods did not see significant changes in hospitalization rates between the two years (*p < 0.05).

Paired analysis results can be seen in Figure 1. There were 4,163 patients who were enrolled in both the 2018 period and the 2019 period. Among patients in that population, 100 unique patients were hospitalized in the 2018 period only, and 96 were hospitalized in the 2019 period only; 22 were hospitalized in both periods. The difference between the percentage of patients who were hospitalized in 2018 (2.93%; 122/4163) and 2019 (2.83%; 118/4163) was not statistically significant (*p* = 0.830; OR = 1.213, 95% CI [0.787, 1.378]). There were 136 total hospitalizations in the 2018 period compared to 122 hospitalizations in the 2019 period, a decrease of 10.29%.

There were 4,952 patients who were enrolled in both the 2019 period and the 2020 period. Among patients in that population, 251 unique patients were hospitalized in the 2019 period only, and 207 were hospitalized in the 2020 period only; 20 were hospitalized in both periods. The difference between the percentage of patients who were hospitalized in 2019 (5.47%; 271/4952) and 2020 (4.58%; 227/4952) was statistically significant (*p* = 0.022; OR = 1.213, 95% CI [1.009, 1.458]). There were 360 total hospitalizations in the 2019 period compared to 280 hospitalizations in the 2020 period, a decrease of 22.22%.

There were 5,138 patients who were enrolled in both the 2020 period and the 2021 period. Among patients in that population, 159 unique patients were hospitalized in the 2020 period only, and 153 were hospitalized in the 2021 period only; 13 were hospitalized in both periods. The difference between the percentage of patients who were hospitalized in 2020 (3.34%; 172/5138) and 2021 (3.23%; 166/5138) was not statistically significant (*p* = 0.777; OR = 1.039, 95% CI [0.832, 1.298]). There were 212 total hospitalizations in the 2020 period compared to 201 hospitalizations in the 2021 period, a decrease of 5.19%.

## Discussion

The results of this study demonstrate a clear correlation between the onset of the COVID-19 pandemic and a decline in mental health-related hospitalization rates. Paired analysis findings show no change in hospitalization rates between two pre-pandemic time periods (2018 and 2019); a decrease in hospitalization rates in the transition between pre-pandemic and pandemic time periods (2019 and 2020, respectively); and no change in hospitalization rates between two pandemic time periods (2020 and 2021). This pattern is reflected in individual year analysis, which found higher hospitalization rates in 2018 and 2019 compared to 2020 and 2021.

The change in modality of psychiatric care, from in-person to virtual, likely contributed to this reduction in hospitalization rates. The increased use of telepsychiatry offers many advantages in patient care. These advantages include fewer barriers to care, increased patient engagement by bringing care directly to their homes and communities, and ease of coordination with patients’ collaterals and caregivers in providing wrap-around care, which together may lead to reduced treatment cost, increased access to quality care, and greater equity in the provision of care (57). Prior research has found telepsychiatry during the COVID-19 pandemic to be satisfactory to providers and effective for patient care; the conclusions drawn here thus complement prior research, and advance the current understanding of the benefits of telepsychiatry to additionally include a reduction in mental health-related hospitalization rates.

It is important to note that mental health-related hospitalization rates serve only as a proxy for efficacy of care, rather than as a specific measure of quality of care such as symptomatology ratings or documented suicide attempts. While this study does not directly address any of these measures, attempted suicide counts in the patient population analyzed here follow the trend seen in hospitalization rates, with 43 in the 2018 time period, 44 in the 2019 time period, 38 in the 2020 time period, and 36 in the 2021 time period (58). This similarity suggests a possible direct causative connection between the use of telepsychiatry and the reduction in mental health-related hospitalization rates.

These findings have important implications for the provision of psychiatric care, but also are relevant for healthcare institutions, as a reduction in inpatient admissions may be associated with significant cost savings. As an example, the Zucker Hillside Hospital inpatient unit has an average length of stay of 16 days and a blended cost per day of about $1,500 per patient; among the 4,952 patients enrolled in both 2019 (in-person care) and 2020 (virtual care), there were 80 fewer hospitalizations in 2020, representing cost savings of approximately $1,920,000.

While telepsychiatry may be associated with enhanced patient care, reduced mental health-related hospitalization rates, and cost savings, it also has disadvantages that must be considered. Concerns include exacerbation of healthcare disparities, such as the risk of worse health outcomes for patients with lower technical literacy, as well as the potential for impaired communication between the patient and the provider. Furthermore, with the expansion of telepsychiatry, the traditional use of catchment areas may become null, such that patients may be receiving care from geographically distant providers. This separation may limit providers’ ability to collaborate with local resources and emergency response teams, thereby obstructing the continuum of care.

Of course, it must be recognized that the increased use of telepsychiatry is unlikely to be the sole determining factor driving reduced mental health-related hospitalization rates. In the context of the COVID-19 pandemic, several other factors must be considered when analyzing these findings. First, during periods of high COVID-19 transmission, many inpatient psychiatric units were closed for admissions due to COVID-positive patients on the unit, thereby leading to a reduced capacity for inpatient hospitalization altogether. There is also the possibility that hospitals were more hesitant to accept new patients, especially patients of lower acuity, during the height of the pandemic, in hopes of reducing COVID-19 transmission; this hesitancy may have resulted in a further reduction in inpatient admissions. Additionally, especially in the beginning of the pandemic, public fear of COVID-19 exposure may have increased patient and family wariness of in-person health encounters, potentially reducing voluntary psychiatric hospitalization. Patients may have been further deterred by policies encouraging mask-wearing while hospitalized, altered visiting hours designed to limit patient exposure to infection, and limited recreational therapy options as compared to usual inpatient programming. In our patient population, it is possible that many patients who previously would have been voluntarily hospitalized were instead absorbed by our hospital’s partial hospitalization program, which converted to telepsychiatry almost immediately at the onset of the pandemic. However, it is unknown for how long following the onset of the COVID-19 pandemic patients would have avoided voluntary hospitalization.

Limitations in our analysis include being unable to account for patients who were enrolled in our hospital’s outpatient programs but were admitted into a different hospital’s inpatient service, and vice versa; however, there are few of these patients, and so they likely do not significantly impact our findings. Additionally, the findings in this study pertain only to one hospital, so caution exists when trying to extrapolate these findings to other hospitals or to the healthcare system at large. Furthermore, as only 2 years’ worth of data were examined prior to and following the onset of the COVID-19 pandemic, there is an inherent risk that the results found are not necessarily representative of the entire pre-pandemic or pandemic periods, but rather are representative only of those few years around the pandemic onset. A final consideration is that our conclusions were reached through examination of retrospective data, leading to results that are reflective of trends within our study population, but that lack the rigor afforded by a study design such as a randomized controlled trial.

To the best of the authors’ knowledge, this is the first study to compare the quality of virtual and in-person psychiatric care using mental health-related hospitalization rates as a metric for patient health. It adds to the growing literature demonstrating the clinical benefits of telepsychiatric care, while also providing psychiatric hospitalization rate data extending several years beyond the onset of the COVID-19 pandemic. The conclusions presented here support the continued use of telepsychiatry in mental healthcare as society transitions into a post-pandemic world, a point of especial relevance now, as pre-COVID-19 restrictive regulations on the use of telepsychiatry are progressively reinstated. Future research should focus on differentiating between the role of the adoption of telepsychiatry and the role of COVID-19-related factors in reducing psychiatric hospitalization rates, while continuing to examine how the quality of telepsychiatry compares to in-patient psychiatric care across various metrics of patient health.

## Data availability statement

The original contributions presented in this study are included in the article/supplementary material, further inquiries can be directed to the corresponding author.

## Author contributions

AC and HL designed the study and organized the raw data for analysis. MJ performed the statistical analysis and aided HL in drawing conclusions from the statistical findings. BL, AC, and HL contributed to the first draft of the manuscript. BL finalized the manuscript for submission. All authors contributed to the editing of the manuscript and preparation for submission.
